# A Randomized, Double-Blind, Placebo-Controlled Investigation of Selenium Supplementation in Women at Elevated Risk for Breast Cancer: Lessons for Re-Emergent Interest in Selenium and Cancer

**DOI:** 10.3390/biomedicines11010049

**Published:** 2022-12-25

**Authors:** Henry J. Thompson, Scot M. Sedlacek, Vanessa K. Fitzgerald, Pamela Wolfe, John N. McGinley

**Affiliations:** 1 Cancer Prevention Laboratory, Colorado State University, Fort Collins, CO 80523, USA; 2 Rocky Mountain Cancer Centers, Denver, CO 80218, USA; 3 Colorado School of Public Health, University of Colorado Anschutz Medical Campus, Aurora, CO 80045, USA

**Keywords:** breast cancer, chemoprevention, selenium, high selenium yeast, antioxidant, oxidative damage, growth factors

## Abstract

Damage to cellular macromolecules such as DNA and lipid, induced via reactive oxygen species, and indicators of cell proliferation potential such as insulin-like growth factor (IGF) metabolic status are intermediate biomarkers of breast cancer risk. Based on reports that selenium status can affect these markers, a randomized, placebo-controlled, double-blind experiment was conducted to investigate the potential of selenium supplementation to modulate breast cancer risk. Using a placebo tablet or a tablet containing 200 μg selenium provided as high-selenium yeast daily for one year, concentrations of the biomarkers in blood or urine were assessed at baseline and after 6 and 12 months of intervention. The selenium intervention used in this study is presumed to mediate its effect via the induction of glutathione peroxidase activity and the consequential impact of the active form of this protein on oxidative damage. We found no evidence to support this hypothesis or to indicate that systemic IGF metabolic status was affected. Critical knowledge gaps must be addressed for the resurgence of interest in selenium and cancer to garner clinical relevance. Those knowledge gaps include the identification of a specific, high-affinity selenium metabolite and the cellular target(s) to which it binds, and the demonstration that the cellular determinant that the selenium metabolite binds plays a critical role in the initiation, promotion, or progression of a specific type of cancer.

## 1. Introduction

Interest in selenium’s involvement in the prevention of chronic diseases, including cancer, originated with studies showing regional differences in soil selenium content, as reviewed in [1,2], and this field is experiencing a significant reemergence in interest [3,4,5,6,7,8,9,10,11,12,13,14,15,16,17]. As reviewed in [18], ecological studies have consistently demonstrated an inverse relationship between selenium exposure and the risk for cancer at several organ sites, including the breast. Those ecological findings have resulted in extensive preclinical investigations that have extended over a period of decades [18,19]. Results from those studies have established a knowledge base regarding the type, amount, frequency, and candidate mechanisms by which selenium exerts inhibitory activity against various cancer types. Most of that evidence indicates that the antioxidant activity of selenium, mediated primarily via different isoforms of glutathione peroxidase, does not account for cancer preventive activity. Despite this, a clinical trial evaluating the impact of high selenium yeast (200 µg selenium/day) on skin cancer (Nutritional Prevention of Cancer, NPC) fueled interest in selenium chemoprevention based on a secondary analysis of that data [20]. That analysis indicated the protective effects of the high selenium yeast intervention against prostate, lung, and colon cancer. To examine these potential beneficial effects, the Selenium and Vitamin E Cancer Prevention Trial (SELECT) was conducted to test the benefit of 200 µg selenium/day given as a seleno-L-methionine supplement for prostate cancer prevention [21,22]. No evidence of benefit was detected [23]. A mechanistic premise of the SELECT trial was based on the established antioxidant effects of selenium and vitamin E despite the preclinical evidence that argued against this mechanism [24,25].

One of the many noteworthy dichotomies in the decades-long investigation of selenium chemoprevention is the large body of preclinical evidence in models for cancer using female animals, particularly for investigating breast cancer. In contrast, limited clinical data on the effect of selenium supplementation has been reported in women at risk for cancer. The ENRICH study reported herein was designed to address this knowledge gap in a population of women at elevated risk for breast cancer as assessed by Gail score and breast density. The amount and type of selenium investigated modeled that reported in the NPC study, i.e., 200 µg selenium/d provided as high selenium yeast. Two intermediate biomarkers of breast cancer risk were selected as endpoints: oxidative damage to DNA and lipid, and cell proliferation potential assessed by insulin-like growth factor status (IGF). Considerable evidence indicates that breast cancer risk is associated with oxidative stress manifest systemically and within breast tissue [26,27]; however, the effect of selenium supplementation on these biomarkers has not been assessed in women. There is also considerable evidence that total cell proliferation exposure in tissue is linked to cancer risk in that tissue [28,29,30,31,32,33,34]. Circulating levels of IGF-1 and its binding protein, IGFBP3, have been linked to breast cell proliferation and breast cancer risk [35,36]. Since there is a small and controversial literature about whether selenium supplementation alters the metabolism of insulin-like growth factor-1 (IGF-1) and its dominant binding protein, IGFBP-3 [37,38,39,40,41,42,43,44], these endpoints were included as study endpoints. The study duration of this randomized, double-blind, placebo-controlled intervention was 12 months with biospecimen collection at baseline, 6, and 12 months during the intervention.

The SELECT trial was conducted during the same timeframe as ENRICH. Because of the distraction created by the negative findings of SELECT, a decision was made to delay the publication of the data reported herein so that it could be more effectively interpreted based on SELECT and the sub-studies designed within that sizeable clinical trial. Publication at this point is prompted by a series of papers reflecting renewed interest in selenium and breast cancer as referenced above [3,4,5,6,7,8,9,10,11,12,13,14,15,16,17].

## 2. Materials and Methods

### 2.1. Study Design

**Figure 1:**
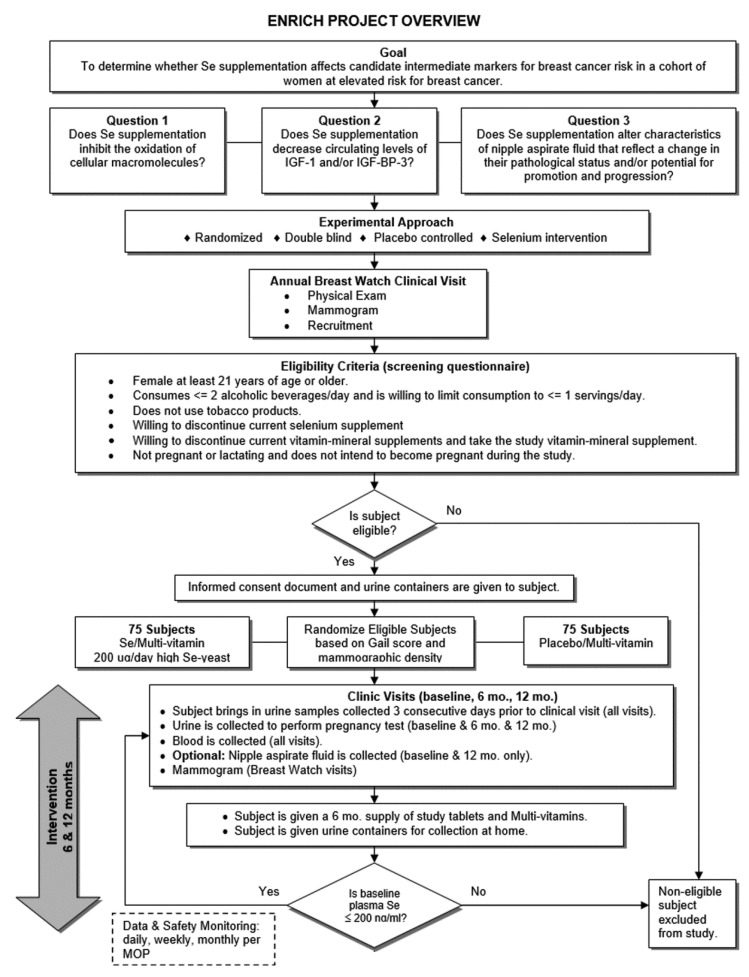
Study Design.

A randomized, double-blind, placebo-controlled intervention was designed to answer two questions: (1) does selenium (Se) supplementation inhibit the oxidation of cellular macromolecules; and (2) does Se supplementation decrease circulating levels of IGF-1 and/or IGFBP-3. Figure 1 summarizes the design of the study. The study began accrual in 2002, and the last subject completed the study protocol in 2006.

### 2.2. Participants

The ENRICH study protocol, approved by the Institutional Review Board, was completely voluntary. One-hundred sixty-two participants, recruited from our high risk BreastWatch clinic, were enrolled. Of these individuals, 111 completed the first clinical visit (baseline, visit 1), 98 completed the 2nd visit, and 94 the 3rd (Table 1). Each participant completed several questionnaires and three physical exams. The initial questionnaires elicited information about demographics, past medical history, supplement use, current medications, and dietary habits. At the first visit, each participant was supplied with multivitamin tablets and study tablets to last until her next visit in approximately 6 months, when taken once daily. Compliance was assessed by measuring plasma selenium levels and pill counts. No significant difference regarding age, Gail score, breast density or randomization group between those who completed the study and those who dropped out. Gail score and breast density were used to stratify subjects to attain balance across the placebo and intervention arms on these two important risk variables.

### 2.3. Sample Collection

The participants also provided samples of blood and urine (2 first void urine samples collected at home and frozen and a fresh sample collected in the clinic) at each of the 3 clinic visits.

#### 2.3.1. Blood

Blood was processed to isolate lymphocytes for DNA isolation and oxidative damage analysis and plasma for analysis of selenium, glutathione peroxidase activity (GPx), and superoxide dismutase activity (SOD). Plasma was stored at −80 °C until analyzed.

#### 2.3.2. Urine

At the same time points that blood was collected, first-void-of-the-morning urine samples were obtained. The decision to use first voids rather than 24 h collections is based largely on this rationale: collecting reliable 24-h urine samples on free-living subjects is difficult, and trying to acquire such samples reduces recruitment potential at the outset and compliance during the study. Collected urine was immediately frozen and, following aliquoting, was stored at −80 °C until analyzed.

### 2.4. Laboratory Analyses

#### 2.4.1. Measurement of Oxidative

DNA Damage by the Comet Assay. The method of Singh was adopted for Comet analysis [45].

#### 2.4.2. Urinary 8-isopostane-F2α

An 8-isoprostane-F2α enzyme immunoassay kit (Assay Designs, Ann Arbor MI, USA) was used. The polyclonal antibody employed by the kit is very specific for 8-isoprostane-F2α.8EPG, and shows minimal cross-reactivity with numerous cyclo oxygenase dependent and independent prostanoids. Prostaglandin F1_α_ does cross-react in this assay, exhibiting ~12% of the activity of 8-isopostane-F2α.

#### 2.4.3. Analyses of IGF-I and IGFBP-3 in Plasma

IGF-I concentrations in plasma was measured by ELISA of an enzymatically amplified “one-step” sandwich-type immunoassay (Diagnostic Systems Laboratories, Webster, TX, USA). The IGF-I concentrations in plasma was calculated from a standard curve plotted as log of the mean absorbance readings for each of the standards along the *y*-axis versus log of the IGF-I concentrations in ng/mL along the *x*-axis, using a linear curve-fit.

#### 2.4.4. IGFBP-3

IGFBP-3 concentrations in plasma was measured by ELISA of an enzymatically amplified “two-step” sandwich-type immunoassay (Diagnostic Systems Laboratories, Webster, TX, USA). The IGFBP-3 concentration in plasma is calculated from a standard curve plotted as the mean absorbance readings for each of the standards along the *y*-axis versus the IGFBP-3 concentrations in ng/mL along the *x*-axis, using a linear-linear curve-fit. Duplicate measurements were performed for all of the samples, as well as standards and internal controls.

#### 2.4.5. Genotyping

Genes and SNPs were selected based on their reported involvement in antioxidant defenses and association with breast cancer [19,46,47,48].

*DNA Isolation* Genomic DNA was isolated from lymphocytes collected during the baseline blood draw using the QIAamp DNA Blood Maxi kit (Qiagen, Germantown, MD, USA) according to the manufacturer’s protocol. Absorbances at 260 nm and 280 nm were utilized to measure DNA concentration and purity using the NanoDrop ND-1000 spectrophotometer (Thermo Fisher Scientific, Waltham, MA, USA). DNA samples were then normalized to 15 ng/μL concentration.

*Real-time PCR SNP genotyping* SNP genotyping was performed by assessing allelic discrimination results of TaqMan assays during real-time PCR. Specific primers and probes were designed using Beacon Designer software (Premier Biosoft Intl, Palo Alto, CA, USA). Probe specificity was confirmed externally by genetic sequencing. Isolated DNA was genotyped for four SNPs of antioxidant genes (listed with their corresponding dbSNP ID): SOD2 (rs1799725), GPX1 (rs1050450), GPX4 (rs713041) and CAT (rs1001179). A list of primers and probes synthesized by IDT (Integrated DNA Technologies, Coralville, CA, USA) and used for genotyping each gene can be found in Supplementary Materials (Table S2).

Real-time TaqMan PCR reactions were set up and run according to the manufacturer’s protocol. Briefly, PCR reactions were assembled in triplicate in optical-grade 96-well plates (Thermo Fisher Scientific, Waltham, MA, USA). Each PCR reaction used 10 μL of 2X iQ Supermix (Bio-Rad, Hercules, CA, USA), 0.5 μL each of forward and reverse primers at 8 μM stock concentration, 0.5 μL each of wild type and mutant probe at 8 μM stock concentration and 8 μL DNA (at 15 ng/μL concentration) for a final volume of 20 μL per reaction. Plates of PCR reactions were run on an iCycler (Bio-Rad, Hercules, CA, USA) under the following conditions: initial melting temperature of 95 °C for 1.5 min followed by 45 cycles of 95 °C for 20 s and 65–70 °C for 1 min. The annealing/elongation temperature varied by primer/probe set as indicated: SOD2-65 °C, GPX1-70 °C, and GPX4/CAT-68 °C. Amplification results were analyzed using iCycler iQ software vs. 3.1 with the Allelic Discrimination function to determine homozygous wild type, heterozygous and homozygous mutant forms of genes from each DNA sample.

#### 2.4.6. Plasma Glutathione Peroxidase (GPx) Activity

Enzyme activity was measured by a modification of the coupled assay procedure described by Lawrence and Burk [45].

#### 2.4.7. Plasma Selenium

Plasma (0.5–1.0 mL) is digested with nitric acid and perchloric acid (3:1) in a borosilicate tube in a hot plate at 100–150 °C. At the end of the digestion, the sample is reacted with hydrochloride acid in 100 °C hot plate for 30 min and incubated with purified 2, 3-diaminonaphthalene containing EDTA and cyclohexane at 60 °C for 30 min. The upper cyclohexane layer is transferred to a fluorescence transparent cuvette and the fluorescence is excited at 385 nm and the emission is read at 525 nm. Atomic absorption standard selenium reference solution was digested and measured at the same time to get a linear standard curve. The result was calculated against the standard curve.

### 2.5. Statistical Methods

Statistical evaluation of the primary outcome measures (levels of urinary 8-EPG, Comet, IGF1, IGFBP3) was done in SAS (version 9.4, Cary, NC, USA) using the mixed procedure to account for repeated measures over time. The fixed effect in the model was selenium intervention (treatment/placebo). Baseline differences among volunteers were evaluated using standard methods (*t*-tests or chi-square) depending on the distribution of the target variable. Full details are presented in the final report to the funding agency [45].

Baseline differences in cohort characteristics across randomization groups were evaluated using a chi-square test for homogeneity of proportions for categorical variables, t-tests or two-group t-tests on the log transform for continuous variables, depending on their distribution.

## 3. Results

### 3.1. Cohort Characteristics

The study participants were predominantly white (96%). Their median age was 49 years (range = 22 to 78). Ninety-seven percent of the participants had more than 12 years of education, 79% reported at least a college degree. They reported consuming an average of 4 ± 2.8 (mean ± SD) servings of vegetables and fruit daily, and had measured BMI of 24 ± 4.5. No baseline characteristics were significantly different by study group. Variables tested were age, race, education, daily servings of vegetables and fruit, plasma selenium, body mass index (BMI), 8-isoprostane-F2α, COMET, IGF-1, IGFBP3, SOD, GPx. GAIL score and breast density were used to stratify the randomization; their means ± SD at baseline were 3.12 ± 1.90 and 54.7% ± 15.9%, respectively.

### 3.2. Compliance Marker Data and Adverse Events

Overall compliance was assessed by pill count was high. Compliance was high: 95% in the placebo group and 96% in the Selenium group. We also measured a biological marker of compliance, i.e., plasma selenium. As reported in Table 2, plasma selenium concentration was elevated in the selenium-supplemented group with very little overlap with the placebo group at either the 6 or 12-month timepoints.

Eleven subjects reported 14 adverse events, 12 mild, and 2 moderate; however, there was no direct link with the selenium supplement (Supplementary Table S1). The adverse event findings are consistent with those reported in NPC and in SELECT.

### 3.3. Outcome Endpoints

#### 3.3.1. Oxidative Damage Biomarkers

Reactive species of oxygen can oxidative damage most macromolecules, but damage of DNA and lipids is considered relevant to breast cancer risk. Relative to DNA, single strand breaks in DNA were assessed by the Comet assay. Magnitive of strand breaks is quantified in arbitary units of damage. As reported in Table 3, damage was not significantly lower in the selenium group at either visit 2 (*p* = 0.51) or visit 3 (*p* = 0.54).

**Figure 2:**
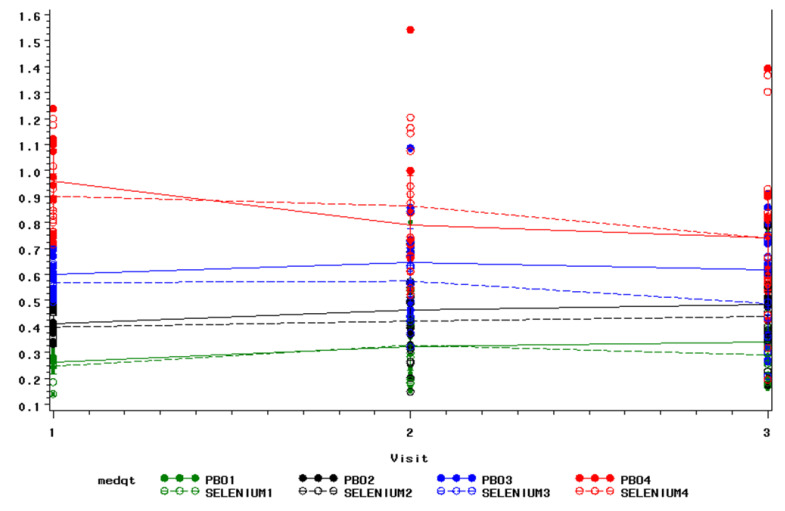
8-isoprostane-F2α by Quartiles.

To assess damage of lipid, excretion of 8-isoprostane-F2α was measured in the urinary since the urinary concentration of this metabolite is considered to be a whole-body index of lipid peroxidation. The concentration of 8-isoprostane-F2α was not significantly lower in the selenium group at either 6-month (*p* = 0.46) or the 12-month timepoint (*p* = 0.70). Because individuals with high levels of lipid peroxidation respond to antioxidant interventions to a greater extent [49], a post hoc analysis (change over time by baseline quartile) was performed. There was no evidence of a differential effect of the intervention by baseline quartile. 8-ISOPGF_α2_ for those in the upper quartile at baseline is marginally lower at 12 months in both groups, while for those in the lower quartile, it is marginally higher in both groups, suggesting regression to the mean (Figure 2).

#### 3.3.2. Proliferative Potential

Circulating concentrations of IGF-1 AND IGFBP-3 are associated with breast cancer risk. Neither measure (Table 3) responded to the selenium supplementation. The differences from the placebo control were not significant at either the 6-month (*p* = 0.68) or the 12-month timepoint (*p* = 0.96).

#### 3.3.3. Exploratory Outcomes

Median GPx was higher in the Selenium intervention group than in the placebo group at 6 months by 8.2% (*p* = 0.04) and at 12 months by 8.7% (*p* = 0.03); the difference between groups at 6 and 12 months for SOD was not significant (*p* = 0.93 and *p* = 0.95, respectively). Genotypes for genes involved in antioxidant defense were also evaluated. Observed frequencies are reported in Table S3. No statistically signficant effect of genotype on study outcome variables was observed.

## 4. Discussion

### 4.1. Overview

Over 25 years, two large selenium chemoprevention studies were conducted in the United States, NPC and SELECT [23,50]. Each study provided a dose of 200 µg selenium/d from an organic source of this essential trace element. Both investigations failed to detect the beneficial effects of selenium supplementation on their primary endpoints, the incidence of skin cancer (NPC) and prostate cancer (SELECT). Moreover, planned secondary analyses in SELECT also failed to detect incident lung or colon cancer effects. ENRICH, the results of which are reported herein, was undertaken in a population of women since women were underrepresented in NPC and were not included in SELECT. ENRICH was designed to address the fundamental question of whether a presumed mechanism of action would be impacted by selenium supplementation, i.e., the oxidative damage of cellular molecules. Because of mixed reports in the scientific literature and documented relevance to breast cancer risk, effects on IGF metabolic status were also evaluated.

### 4.2. The Form and Dose of Selenium

The historical context surrounding the design and launch of the NPC trial is important. At that time, it was well recognized that the activity of glutathione peroxidase was saturated at levels of intake of <100 µg selenium per day irrespective of source [51]. It was also understood that the range between meeting nutritional requirements and manifestation of toxicity was narrow [52,53,54]. There was also concern that whole body selenium load would be increased if an organic selenium source was used since mammalian protein synthesis fails to distinguish between methionine and seleno-L-methionine, a predominant form of selenium in selenium enriched brewer’s yeast. Excessive accumulation of seleno-amino acids could result in unintentional toxicity if excessive body protein catabolism occurred [1]. Despite these issues and concerns, NPC was granted an IND for high selenium yeast at a dose providing 200 µg/day. One can argue that given these facts there was no clear mechanistic rationale for NPC. In the selenium arms of SELECT, the organic source of selenium was seleno-L-methionine and the fact that selenium and vitamin E were combined in one arm of the study demonstrates that a mechanistic focus was on antioxidant activity. ENRICH followed the NPC design, but with two specific mechanistic hypotheses: selenium mediated protection against oxidative cellular damage and selenium mediated attenuation of proliferative drive affected by growth factors. To our knowledge, no sub studies in either NPC or SELECT evaluated these endpoints.

### 4.3. Positioning Effects of Selenium in a Mechanistic Framework

While debated extensively, publication of the concept that cell proliferation in stem cell populations within a tissue is a major driver of the occurrence of mutations that result in the development of cancer is instructive [29,34]. The refinement of this concept to encompass the mutations that are heredity and those that emerge from environmental exposures further enhances the contextual framework for formulating expectations for when selenium would be expected to protect against cancer [30,32]. The two most likely scenarios are: (1) reduced oxidative damage over time leading to reduced mutational load; and (2) a reduced cumulative proliferative load on stem cell populations in the breast. ENRICH provides a snapshot view of both scenarios.

The effects of the selenium intervention used in ENRICH are generally presumed to be mediated via the induction of glutathione peroxidase activity and the consequential effect of the active form of this protein on antioxidant status. Although selenium supplementation did induce a nominal increase in glutathione peroxidase activity (Table 3), the change was not associated with a decrease in the oxidation of either DNA or lipid. It is noteworthy that a lack of effect is consistent with work in men [55], but differs from the correlation among serum selenium, selenoprotein P, and glutathione peroxidase activity, three markers of selenium status, that have been reported to identify patients at high risk for poor prognosis at the time of breast cancer diagnosis [4]. Nonetheless, we found no evidence to support this hypothesis, i.e., the first scenario mentioned in the preceding paragraph. Relative to the second scenario, an effect on cumulative proliferative load, there is a small and controversial literature about whether selenium supplementation alters the metabolism of insulin-like growth factor-1 (IGF-1) and its dominant binding protein, IGFBP-3 [37,38,39,40,41,42,43,44]. Elevated IGF-1 and IGBP3 have both recently been affirmed to be risk factors for breast cancer [35,36]. However, we found no evidence to support the hypothesis that IGF-1 or IGFBP-3 metabolism was affected by the selenium intervention. Relative to hereditary status, individuals in ENRICH were recruited from a population at increased risk for breast cancer based on their Gail score or increased breast density. When statistical models included these variables, neither was associated with a statistically significant effect on study outcomes. Similarly, the differences in the genotypes for genes involved in antioxidant defense and that have been associated with breast cancer had no significant impact on study outcomes [19,46,47,48].

### 4.4. Lessons from the Preclinical Literature and a Cautionary Note

The preclinical literature on selenium and cancer is extensive. The earliest studies in breast cancer were conducted in a model with viral etiology, and chronologically, those studies were followed by investigations using chemically induced breast cancer models. Those studies: (1) provided little evidence for a role of glutathione peroxidase induction in the mediation of cancer inhibitory activity, and (2) did not attempt to contrast effects in virally versus chemically induced cancer models. What the chemically induced models did do was: (1) indicate that the cancer inhibitory species of selenium was a product of the final stages of selenium’s metabolism leading to its elimination from the body, and (2) identify inhibition of proliferation and induction of apoptosis as cellular mediators of protective activity. However, efforts to identify both a specific selenium metabolite and its specific cellular target that played a causal role in the development of breast cancer were never achieved. Rather, in retrospect, a careful inspection of the time course of develops in the preclinical investigations of causal mechanisms reveals that those efforts stop with a dramatic shift in the field to attention on NPC and SELECT. This was unfortunate because the lack of identification of causal mechanisms remains a major obstacle in further pursuit of this line of investigation in the clinic.

### 4.5. Taking a Fresh Look at Ecological Evidence

The lessons that can be learned from the natural experiment of regional differences in environmental selenium levels, selenium intake, and disease etiology have never been fully considered in the selenium and cancer field. In part this is because the importance of the immune system’s involvement in cancer prevention and control has only been clearly demonstrated over the last 20 years; whereas, the seminal preclinical and clinical studies of selenium occurred prior to that time. To the point, one of the strongest impacts of improving selenium status in low selenium regions in China was reduced occurrence of Keshan disease, a disease of viral etiology [56]. More recently, evidence has arisen of impacts of selenium on morbidity and mortality in response to COVID-19 [18,57,58,59,60]. From a co-translational perspective, many years ago, evidence was advanced that selenium impacts disease of viral etiology and that the immune system is involved. The lesson here is twofold: (1) this is a minimally investigated area that merits attention, but (2) the data is much too limited for other than exploratory analyses. If, however, a concrete role for a selenometabolite and its cell target can be demonstrated on immune function, the focus of analysis should be expanded from cancer prevention to include cancer control. It is also possible that selenium induced effects could be mediated by microorganisms in the gut [61], but such secondary effects would need to be rigorously probed to identify specific causal mechanisms.

### 4.6. As Good as It Gets?

The role of selenium as an essential dietary trace element is clear. If nothing else, the clinical trials represented by NPC and SELECT have elucidated that higher doses of selenium than were at one time considered safe are well tolerated. In addition, new selenoproteins and selenium binding proteins were identified, and greater insights into the cellular metabolism of this trace element were achieved. That may be as good as it gets and that is clearly an acceptable outcome. However, with the re-emergent interest in selenium and cancer, it would be prudent for investigators to plow new ground rather than to return to the same ideas that dominated the field in the period from 1980 to 2005.

## 5. Conclusions

Selenium is both a nutritionally essential trace element and a toxicant depending on the type and amount of the element that is consumed [1]. A substantial body of mechanistic data supports these two facts. As reported herein, the hypothesis that selenium supplementation in replete individuals improves their antioxidant status as assessed by systemic levels of oxidative cellular damage or that it alters proliferative drive by affecting IGF metabolism is not supported. These observations are consistent with the lack of effect observed in NPC and SELECT. Critical knowledge gaps must be filled for the resurgence of interest in selenium and cancer to garner clinical relevance. These critical knowledge deficits are simply stated but will be difficult to fill: the identification of a specific, high-affinity selenium metabolite and the cellular target(s) to which it binds and the demonstration that the cellular determinant to which the seleno-metabolite binds plays a critical role in the initiation, promotion, or progression of a specific type of cancer. Currently, there is no strong justification for proposing an intervention trial in women at risk for breast cancer.

## Figures and Tables

**Table 1:** Participants who completed the study by visit and study arm.

	Enrollment	Visit 1 (Baseline)	Visit 2	Visit 3	Dropout Rate ^1^
Selenium	134	55	48	47	15%
Placebo		56	50	47	16%
Not Randomized	28				
Total	162	111	98	94	15%

^1^ Dropout rate computed based the subjects who completed their first clinic visit.

**Table 2:** Plasma Selenium Levels at baseline and 6 and 12 months by study arm.

	Treatment Group	Baseline (*n* = 111)	6 Months (*n* = 98)	12 Months (*n* = 93)
Plasma Se	Selenium	122.28 ± 13.99	195.60 ± 29.35	203.00 ± 34.11
	Placebo	126.02 ± 12.87	132.30 ± 14.58	130.78 ± 14.66

Values are means ± SD.

**Table 3:** Outcome Measures at Baseline and Follow-up.

Outcome Measure ^1^	Treatment Group	Baseline (*n* = 93)	6 Months (*n* = 93)	*p*	12 Months (*n* = 93)	*p*
8-isoprostane-F2α (pg/µg creatinine)	Se	0.49 (0.31, 0.72)	0.54 (0.38, 0.73)	0.46	0.43 (0.30, 0.66)	0.70
	Placebo		0.49 (0.34, 0.69)		0.50 (0.35, 0.75)	
DNA damage (arbitrary units/cell)	Se	46.6 (35.2, 55.00)	45.4 (39.1, 52.1)	0.51	36.2 (22.8, 49.5)	0.54
	Placebo		50.3 (39.9, 57.2)		31.4 (30.7, 36.4)	
IGF1 (ng/mL)	Se	238 (184, 305)	266 (193, 306)	0.76	237 (181, 307)	0.81
	Placebo		242 (176, 295)		220 (176, 298)	
IGFBP3 (ng/mLl)	Se	4527 (3954, 5388)	4402 (3819, 5332)	0.68	4213 (3762, 4993)	0.96
	Placebo		4251 (3992, 5233)		4188 (3857, 5098)	
Explanatory Outcomes						
SOD (nmol/min/mL)	Se	3.87 (3.08, 4.67)	3.95 (3.24, 4.98)	0.98	3.77 (3.27, 4.73)	0.99
	Placebo		4.23 (3.26, 4.82)		4.17 (3.51, 4.82)	
GPx (nmol/min/mL)	Se	103 (83, 115)	121 (105, 138)	0.04	122 (112, 134	0.03
	Placebo		112 (91, 128)		112 (97, 134)	

^1^ Values are medians (pctl 25, pctl 75).

## Supplementary Materials

The following supporting information can be downloaded at: https://www.mdpi.com/article/10.3390/biomedicines11010049/s1, Table S1: Adverse events; Table S2: Primer sequences; Table S3: Genotype frequencies.

## References

[1] Rayman M.P. (2020). Selenium intake, status, and health: A complex relationship. Hormones.

[2] Zhang J., Taylor E.W., Bennett K., Saad R., Rayman M.P. (2020). Association between regional selenium status and reported outcome of COVID-19 cases in China. Am. J. Clin. Nutr..

[3] Bengtsson Y., Demircan K., Rosendahl A.H., Borgquist S., Sandsveden M., Manjer J. (2022). Zinc and Breast Cancer Survival: A Prospective Cohort Study of Dietary Intake and Serum Levels. Nutrients.

[4] Demircan K., Bengtsson Y., Sun Q., Brange A., Vallon-Christersson J., Rijntjes E., Malmberg M., Saal L.H., Ryden L., Borg A. (2021). Serum selenium, selenoprotein P and glutathione peroxidase 3 as predictors of mortality and recurrence following breast cancer diagnosis: A multicentre cohort study. Redox Biol..

[5] Demircan K., Chillon T.S., Sun Q., Heller R.A., Klingenberg G.J., Hirschbil-Bremer I.M., Seemann P., Diegmann J., Bachmann M., Moghaddam A. (2022). Humoral immune response to COVID-19 mRNA vaccination in relation to selenium status. Redox Biol..

[6] Demircan K., Sun Q., Bengtsson Y., Seemann P., Vallon-Christersson J., Malmberg M., Saal L.H., Ryden L., Minich W.B., Borg A. (2022). Autoimmunity to selenoprotein P predicts breast cancer recurrence. Redox Biol..

[7] Zou B., Xiong Z., He L., Chen T. (2022). Reversing breast cancer bone metastasis by metal organic framework-capped nanotherapeutics via suppressing osteoclastogenesis. Biomaterials.

[8] Zigrossi A., Hong L.K., Ekyalongo R.C., Cruz-Alvarez C., Gornick E., Diamond A.M., Kastrati I. (2022). SELENOF is a new tumor suppressor in breast cancer. Oncogene.

[9] Vahid F., Rahmani W., Khodabakhshi A., Davoodi S.H. (2022). Associated between Dietary Antioxidant Index (DAI) and Odds of Breast Cancer and Correlation between DAI with Pathobiological Markers: Hospital-Based Incidence Case-Control Study. J. Am. Nutr. Assoc..

[10] Sohouli M.H., Baniasadi M., Hernandez-Ruiz A., Melekoglu E., Zendehdel M., Jose Soto-Mendez M., Akbari A., Zarrati M. (2022). Adherence to Oxidative Balance Scores is Associated with a Reduced Risk of Breast Cancer; A Case-Control Study. Nutr. Cancer.

[11] Radomska D., Czarnomysy R., Szymanowska A., Radomski D., Dominguez-Alvarez E., Bielawska A., Bielawski K. (2022). Novel Selenoesters as a Potential Tool in Triple-Negative Breast Cancer Treatment. Cancers.

[12] Martinez-Esquivias F., Gutierrez-Angulo M., Perez-Larios A., Sanchez-Burgos J.A., Becerra-Ruiz J.S., Guzman-Flores J.M. (2022). Anticancer Activity of Selenium Nanoparticles In Vitro Studies. Anticancer Agents Med. Chem..

[13] Zhu X., Pan D., Wang N., Wang S., Sun G. (2021). Relationship Between Selenium in Human Tissues and Breast Cancer: A Meta-analysis Based on Case-Control Studies. Biol. Trace Elem. Res..

[14] Woo J., Kim J.B., Cho T., Yoo E.H., Moon B.I., Kwon H., Lim W. (2021). Selenium inhibits growth of trastuzumab-resistant human breast cancer cells via downregulation of Akt and beclin-1. PLoS ONE.

[15] Szwiec M., Marciniak W., Derkacz R., Huzarski T., Gronwald J., Cybulski C., Debniak T., Jakubowska A., Lener M., Falco M. (2021). Serum Selenium Level Predicts 10-Year Survival after Breast Cancer. Nutrients.

[16] Soltani L., Darbemamieh M. (2021). Anti-proliferative, apoptotic potential of synthesized selenium nanoparticles against breast cancer cell line (MCF7). Nucleosides Nucleotides Nucleic Acids.

[17] Lee H., Lee B., Kim Y., Min S., Yang E., Lee S. (2021). Effects of Sodium Selenite Injection on Serum Metabolic Profiles in Women Diagnosed with Breast Cancer-Related Lymphedema-Secondary Analysis of a Randomized Placebo-Controlled Trial Using Global Metabolomics. Nutrients.

[18] Rataan A.O., Geary S.M., Zakharia Y., Rustum Y.M., Salem A.K. (2022). Potential Role of Selenium in the Treatment of Cancer and Viral Infections. Int. J. Mol. Sci..

[19] Rayman M.P. (2005). Selenium in cancer prevention: A review of the evidence and mechanism of action. Proc. Nutr. Soc..

[20] Clark L.C., Combs G.F., Turnbull B.W., Slate E.H., Chalker D.K., Chow J., Davis L.S., Glover R.A., Graham G.F., Gross E.G. (1996). Effects of selenium supplementation for cancer prevention in patients with carcinoma of the skin. A randomized controlled trial. Nutritional Prevention of Cancer Study Group. JAMA.

[21] Klein E.A., Thompson I.M., Lippman S.M., Goodman P.J., Albanes D., Taylor P.R., Coltman C. (2003). SELECT: The selenium and vitamin E cancer prevention trial. Urol. Oncol..

[22] Klein E.A., Thompson I.M., Lippman S.M., Goodman P.J., Albanes D., Taylor P.R., Coltman C. (2001). SELECT: The next prostate cancer prevention trial. Selenum and Vitamin E Cancer Prevention Trial. J. Urol..

[23] Lippman S.M., Klein E.A., Goodman P.J., Lucia M.S., Thompson I.M., Ford L.G., Parnes H.L., Minasian L.M., Gaziano J.M., Hartline J.A. (2009). Effect of selenium and vitamin E on risk of prostate cancer and other cancers: The Selenium and Vitamin E Cancer Prevention Trial (SELECT). JAMA.

[24] Klein E.A., Thompson I.M., Lippman S.M., Goodman P.J., Albanes D., Taylor P.R., Coltman C. (2000). SELECT: The Selenium and Vitamin E Cancer Prevention Trial: Rationale and design. Prostate Cancer Prostatic Dis..

[25] Lippman S.M., Goodman P.J., Klein E.A., Parnes H.L., Thompson I.M., Kristal A.R., Santella R.M., Probstfield J.L., Moinpour C.M., Albanes D. (2005). Designing the Selenium and Vitamin E Cancer Prevention Trial (SELECT). J. Natl. Cancer Inst..

[26] Lee J.D., Cai Q., Shu X.O., Nechuta S.J. (2017). The Role of Biomarkers of Oxidative Stress in Breast Cancer Risk and Prognosis: A Systematic Review of the Epidemiologic Literature. J. Womens Health.

[27] Erdmann N.J., Harrington L.A., Martin L.J. (2017). Mammographic density, blood telomere length and lipid peroxidation. Sci. Rep..

[28] Chan T.A., Wang Z., Dang L.H., Vogelstein B., Kinzler K.W. (2002). Targeted inactivation of CTNNB1 reveals unexpected effects of beta-catenin mutation. Proc. Natl. Acad. Sci. USA.

[29] Tomasetti C., Durrett R., Kimmel M., Lambert A., Parmigiani G., Zauber A., Vogelstein B. (2017). Role of stem-cell divisions in cancer risk. Nature.

[30] Tomasetti C., Li L., Vogelstein B. (2017). Stem cell divisions, somatic mutations, cancer etiology, and cancer prevention. Science.

[31] Tomasetti C., Poling J., Roberts N.J., London N.R., Pittman M.E., Haffner M.C., Rizzo A., Baras A., Karim B., Kim A. (2019). Cell division rates decrease with age, providing a potential explanation for the age-dependent deceleration in cancer incidence. Proc. Natl. Acad. Sci. USA.

[32] Tomasetti C., Vogelstein B. (2017). On the slope of the regression between stem cell divisions and cancer risk, and the lack of correlation between stem cell divisions and environmental factors-associated cancer risk. PLoS ONE.

[33] Tomasetti C., Vogelstein B. (2015). Cancer risk: Role of environment-response. Science.

[34] Tomasetti C., Vogelstein B. (2015). Cancer etiology. Variation in cancer risk among tissues can be explained by the number of stem cell divisions. Science.

[35] Murphy N., Knuppel A., Papadimitriou N., Martin R.M., Tsilidis K.K., Smith-Byrne K., Fensom G., Perez-Cornago A., Travis R.C., Key T.J. (2020). Insulin-like growth factor-1, insulin-like growth factor-binding protein-3, and breast cancer risk: Observational and Mendelian randomization analyses with approximately 430 000 women. Ann. Oncol..

[36] Costa-Silva D.R., Barros-Oliveira M.D.C., Silva B.B.D. (2021). Systematic review of insulin-like growth factor 1 gene expression in women with breast cancer. Rev. Assoc. Med. Bras..

[37] Maggio M., Ceda G.P., Lauretani F., Bandinelli S., Dall’Aglio E., Guralnik J.M., Paolisso G., Semba R.D., Nouvenne A., Borghi L. (2010). Association of plasma selenium concentrations with total IGF-1 among older community-dwelling adults: The InCHIANTI study. Clin. Nutr..

[38] Liu J.G., Zhao H.J., Liu Y.J., Wang X.L. (2006). Effect of selenium-enriched malt on hepatocarcinogenesis, paraneoplastic syndrome and the hormones regulating blood glucose in rats treated by diethylnitrosamine. Life Sci..

[39] Kristal A.R., King I.B., Albanes D., Pollak M.N., Stanzyk F.Z., Santella R.M., Hoque A. (2005). Centralized blood processing for the selenium and vitamin E cancer prevention trial: Effects of delayed processing on carotenoids, tocopherols, insulin-like growth factor-I, insulin-like growth factor binding protein 3, steroid hormones, and lymphocyte viability. Cancer Epidemiol. Biomark Prev..

[40] Aydin K., Bideci A., Kendirci M., Cinaz P., Kurtoglu S. (2002). Insulin-like growth factor-I and insulin-like growth factor binding protein-3 levels of children living in an iodine- and selenium-deficient endemic goiter area. Biol. Trace Elem. Res..

[41] Moreno-Reyes R., Egrise D., Neve J., Pasteels J.L., Schoutens A. (2001). Selenium deficiency-induced growth retardation is associated with an impaired bone metabolism and osteopenia. J. Bone Miner. Res..

[42] Thorlacius-Ussing O., Flyvbjerg A., Orskov H. (1988). Growth in young rats after termination of sodium selenite exposure: Studies of growth hormone and somatomedin C. Toxicology.

[43] Thorlacius-Ussing O., Flyvbjerg A., Jorgensen K.D., Orskov H. (1988). Growth hormone restores normal growth in selenium-treated rats without increase in circulating somatomedin C. Acta Endocrinol..

[44] Thorlacius-Ussing O., Flyvbjerg A., Esmann J. (1987). Evidence that selenium induces growth retardation through reduced growth hormone and somatomedin C production. Endocrinology.

[45] Thompson H.J. Selenium and Breast Cancer Chemoprevention. https://archive.org/details/DTIC_ADA4112872007.

[46] Mathers J.C., Hesketh J.E. (2007). The biological revolution: Understanding the impact of SNPs on diet-cancer interrelationships. J. Nutr..

[47] Oestergaard M.Z., Tyrer J., Cebrian A., Shah M., Dunning A.M., Ponder B.A., Easton D.F., Pharoah P.D. (2006). Interactions between genes involved in the antioxidant defence system and breast cancer risk. Br. J. Cancer.

[48] Udler M., Maia A.T., Cebrian A., Brown C., Greenberg D., Shah M., Caldas C., Dunning A., Easton D., Ponder B. (2007). Common germline genetic variation in antioxidant defense genes and survival after diagnosis of breast cancer. J. Clin. Oncol..

[49] Thompson H.J., Heimendinger J., Sedlacek S., Haegele A., Diker A., O’Neill C., Meinecke B., Wolfe P., Zhu Z., Jiang W. (2005). 8-Isoprostane F2alpha excretion is reduced in women by increased vegetable and fruit intake. Am. J. Clin. Nutr..

[50] Clark L.C., Dalkin B., Krongrad A., Combs G.F., Turnbull B.W., Slate E.H., Witherington R., Herlong J.H., Janosko E., Carpenter D. (1998). Decreased incidence of prostate cancer with selenium supplementation: Results of a double-blind cancer prevention trial. Br. J. Urol..

[51] Whanger P.D., Beilstein M.A., Thomson C.D., Robinson M.F., Howe M. (1988). Blood selenium and glutathione peroxidase activity of populations in New Zealand, Oregon, and South Dakota. Faseb. J..

[52] Frost D.V. (1972). The two faces of selenium--can selenophobia be cured?. CRC Crit. Rev. Toxicol..

[53] Harr J.R., Muth O.H. (1972). Selenium poisoning in domestic animals and its relationship to man. Clin. Toxicol.

[54] Lo M.T., Sandi E. (1980). Selenium: Occurrence in foods and its toxicological significance--a review. J. Environ. Pathol. Toxicol..

[55] Richie J.P., Das A., Calcagnotto A.M., Sinha R., Neidig W., Liao J., Lengerich E.J., Berg A., Hartman T.J., Ciccarella A. (2014). Comparative effects of two different forms of selenium on oxidative stress biomarkers in healthy men: A randomized clinical trial. Cancer Prev. Res..

[56] Hoffmann P.R., Berry M.J. (2008). The influence of selenium on immune responses. Mol. Nutr. Food Res..

[57] Fath M.K., Naderi M., Hamzavi H., Ganji M., Shabani S., Ghahroodi F.N., Khalesi B., Pourzardosht N., Hashemi Z.S., Khalili S. (2022). Molecular mechanisms and therapeutic effects of different vitamins and minerals in COVID-19 patients. J. Trace Elem. Med. Biol..

[58] Majeed M., Nagabhushanam K., Prakasan P., Mundkur L. (2022). Can Selenium Reduce the Susceptibility and Severity of SARS-CoV-2?-A Comprehensive Review. Int. J. Mol. Sci..

[59] Skesters A., Kustovs D., Lece A., Moreino E., Petrosina E., Rainsford K.D. (2022). Selenium, selenoprotein P, and oxidative stress levels in SARS-CoV-2 patients during illness and recovery. Inflammopharmacology.

[60] Pedrosa L.F.C., Barros A., Leite-Lais L. (2022). Nutritional risk of vitamin D, vitamin C, zinc, and selenium deficiency on risk and clinical outcomes of COVID-19: A narrative review. Clin. Nutr. ESPEN.

[61] Maitiniyazi G., Cao X., Chen Y., Zhang R., Liu Y., Li Z., Gu D., Li T., Xia S. (2022). Impact of Gut Microbiota on the Association between Diet and Depressive Symptoms in Breast Cancer. Nutrients.

