# Supplementary material for: A Randomized, Double-Blind, Placebo-Controlled Investigation of Selenium Supplementation in Women at Elevated Risk for Breast Cancer: Lessons for Re-Emergent Interest in Selenium and Cancer
Source: Biomedicines. 2022 Dec 25;11(1):49. doi: 10.3390/biomedicines11010049 (PMC9855926; doi:10.3390/biomedicines11010049)
Supplement: Supplementary file 1 [file biomedicines-11-00049-s001.zip › biomedicines-2057862-supplementary.pdf]

biomedicines

MDPI

Supplementary Materials

Table S1. Adverse events

Adverse Event Type	Severity	Relation To Study Tablets	Details
Unanticipated	Mild	Possible	cramps, diarrhea, bloating
Unanticipated	Mild	Possible	Stomach discomfort
Unanticipated	Mild	Possible	Stomach discomfort
Unanticipated	Mild	Not Related	breast pain – follow up with oncologist.
Unanticipated	Mild	Unlikely	Sleeplessness
Unanticipated	Mild	Unlikely	Gastric reflux
Unanticipated	Moderate	Possible	Vomiting, stomach discomfort
Unanticipated	Mild	Possible	stomach discomfort
Unanticipated	Mild	Unlikely	"body vibrations" – follow up with primary care physician
Unanticipated	Mild	Not Related	Increased bilirubin - may be due to history of Crones disease

Table S2. Primer sequences

Gene	Primer Direction	Primer Sequence	Probe Type	Probe Sequence
SOD2	Forward	5'-GGCTGTGCTTTCTCGTCTTCA-3'	Wild Type	5'-FAM-TACCCCAAA**G**CCGGAGCCAC-3BHQ1-3′
	Reverse	5'-GCTGTGCTTCTGCCTGGAG-3'	Mutant	5'-HEX-ATACCCCAAA**A**CCGGAGCCAG-3BHQ1-3′
GPX1	Forward	5'-CCATTGACATCGAGCCTGACATC-3'	Wild Type	5'-FAM-AGGCACAGCTG**G**GCCCTTGAGAC-3BHQ1-3′
	Reverse	5'-GCCAAGCAGCCGGGGTAG-3'	Mutant	5'-HEX-AGGCACAGCTG**A**GCCCTTGAGAC-3BHQ1-3′
GPX4	Forward	5′-GGACCTGCCCCACTATTTCTAGC-3′	Wild Type	5'-FAM-TGCCCACGCCCT**T**GGAGCCTT-3BHQ1-3′
	Reverse	5′-TGCACGCTGGATTTTTCGGGT-3′	Mutant	5'-TET-TGCCCACGCCCT**C**GGAGCCTT-3BHQ1-3′
CAT	Forward	5'-GGCGGCCTGAAGGATGCT-3'	Wild Type	5'-FAM-TGCCCGG**G**ATAGCCGAACCC-3BHQ1-3'
	Reverse	5'-GCCCAGCAATTGGAGAGCCT-3'	Mutant	5'-TET-TGCCCGG**A**ATAGCCGAACCC-3BHQ1-3′

Table S3. Results of Allelic Discrimination analysis by Gene SNP

Gene	dbSNP ID	Genotype	Subjects	Observed Allelic Frequencies
SOD2	rs1799725	CC	32	0.34
		CT	37	0.39
		TT	25	0.27
GPX1	rs1050450	CC	40	0.43
		CT	47	0.50
		TT	7	0.07
GPX4	rs713041	TT	15	0.16
		TC	49	0.52
		CC	30	0.32
CAT	rs1001179	CC	57	0.61
		CT	33	0.35
		TT	4	0.04
